# Gene loss, genome rearrangement, and accelerated substitution rates in plastid genome of *Hypericum ascyron* (Hypericaceae)

**DOI:** 10.1186/s12870-022-03515-x

**Published:** 2022-03-23

**Authors:** Sivagami-Jean Claude, Seongjun Park, SeonJoo Park

**Affiliations:** 1 Department of Life Sciences, Yeungnam University, Gyeongsan, Gyeongbuk 38541 South Korea; 2 Institute of Natural Science, Yeungnam University, Gyeongsan, Gyeongbuk 38541 South Korea

**Keywords:** Gene transfer, Gene substitution, Plastome, Rearrangement, Inversion, *matK*

## Abstract

**Background:**

Comparative genomic analysis exhibits dynamic evolution of plastid genome (plastome) in the clusioid clade of Malpighiales, which comprise five families, including multiple inversions and gene losses. Little is known about the plastome evolution in Hypericaceae, a large family in the clade. Only the plastome of one species, *Cratoxylum cochinchinense,* has been published.

**Results:**

We generated a complete plastome sequence for *Hypericum ascyron*, providing the first complete plastome from the tribe Hypericeae (Hypericaceae). The *H. ascyron* plastome exhibits dynamic changes in gene and intron content, structure, and sequence divergence compared to the *C. cochinchinense* plastome from the tribe Cratoxyleae (Hypericaceae). Transcriptome data determined the evolutionary fate of the missing plastid genes *infA*, *rps7*, *rps16*, *rpl23*, and *rpl32* in *H. ascyron*. Putative functional transfers of *infA*, *rps7,* and *rpl32* were detected to the nucleus, whereas *rps16* and *rpl23* were substituted by nuclear-encoded homologs. The plastid *rpl32* was integrated into the nuclear-encoded *SODcp* gene. Our findings suggested that the transferred *rpl32* had undergone subfunctionalization by duplication rather than alternative splicing. The *H. ascyron* plastome rearrangements involved seven inversions, at least three inverted repeat (IR) boundary shifts, which generated gene relocations and duplications. Accelerated substitution rates of plastid genes were observed in the *H. ascyron* plastome compared with that of *C. cochinchinense* plastid genes. The higher substitution rates in the *accD* and *clpP* were correlated with structural change, including a large insertion of amino acids and losses of two introns, respectively. In addition, we found evidence of positive selection of the *clpP*, *matK*, and *rps3* genes in the three branches related to *H. ascyron*. In particular, the *matK* gene was repeatedly under selection within the family Hypericaceae. Selective pressure in the *H. ascyron matK* gene was associated with the loss of *trnK*-UUU and relocation into the IR region.

**Conclusions:**

The *Hypericum ascyron* plastome sequence provides valuable information for improving the understanding of plastome evolution among the clusioid of the Malpighiales. Evidence for intracellular gene transfer from the plastid to the nucleus was detected in the nuclear transcriptome, providing insight into the evolutionary fate of plastid genes in Hypericaceae.

**Supplementary Information:**

The online version contains supplementary material available at 10.1186/s12870-022-03515-x.

## Background

Plastids are one of energy-producing eukaryotic organelles that originate from cyanobacterial-like endosymbionts. Plastids contain their own genome, which is highly reduced compared to the ancestral genome due to massive losses in genes or their transfers into the nuclear genome [1]. Moreover, gene transfer from plastids to the nucleus is an ongoing process [2, 3]. Among angiosperms, plastid genomes (plastomes) are highly conserved in structure, mainly encoding for the photosynthetic or transcription apparatus of the organelles and their own replication apparatus. The plastomes have a quadripartite structure with large and small single copy (LSC and SSC, respectively) regions separated by two inverted repeat (IR) regions, ranging from 120 to 170 kb in length [4]. However, several genome rearrangements have been found in Asteraceae [5], Campanulaceae [6], Fabaceae [7], Geraniaceae [8], Oleaceae [9], Papaveraceae [10], Plantaginaceae [11, 12], and Poaceae [13] across angiosperm plastomes. Previous studies have suggested that plastome rearrangements are correlated with the number and size of repeats [7, 8, 14]. IR expansions and contractions also contribute to plastome rearrangements and gene content variations [10, 14, 15]. Although gene and intron contents are generally conserved in the plastomes, comparative analyses of these contents in angiosperm plastomes showed a variation in the losses of 27 protein-encoding genes and 8 introns [16]. Among them, functional replacements of plastid genes by gene transfer to the nucleus or by nuclear homolog substitution have been documented for only 11 plastid genes: *accD* [17–23], *infA* [24, 25], *rpl20* [26], *rpl22* [26–28], *rpl23* [29, 30], *rpl32* [24, 25, 31–33], *rps7* [26], *rps15* [10], *rps16* [25, 33–35], *ycf1* and *ycf2* [26].

The family Hypericaceae comprises of approximately 700 species in 9 genera, which belong to the clusioid of the Malpighiales [36]. Hypericaceae has been classified into three tribes: Cratoxyleae, Hypericeae, and Vismieae. The relationships between the genera within three tribes are unclear. *Hypericum*, a member of the tribe Hypericeae, is the largest genus in the family with more than 490 species and has a cosmopolitan distribution [37]. Some species in this genus are economically and medically important plants that produce different kinds of naphthodianthrones (especially hypericin), acylphloroglucinol derivatives, and flavonoid compounds [38], which are used as depression medications and painkillers [39, 40]. Despite the many therapeutic applications of *Hypericum* spp., a natural product genomics approach is limited. Only the one whole genome sequence data of *H. perforatum* have been reported [41], and those for complete plastid and mitochondrial genomes belonging to this genus are yet unreported.

In the order Malpighiales, which comprises of 16,000 species in 716 genera and 36 families, approximately 236 plastomes from 69 genera have been sequenced (National Center for Biotechnology Information; NCBI, accessed on June 11, 2021), and the average plastome size and GC content for the order were 155.6 kb and 36.5%, respectively. Previous studies of comparative analyses have revealed that the plastomes are highly conserved in structural organization with a few exceptions (Euphorbiaceae, Passifloraceae, and Podostemaceae) [42–45]. Sequencing data of the plastomes from this order have also provided excellent examples of gene and intron loss. The evolutionary fate of these missing plastid genes showed that they were functionally replaced by gene transfer to the nucleus or by gene substitution of a nuclear gene. For example, transfers of *infA* and *rpl32* and substitution of *rps16* are well-characterized examples in Malpighiales [26, 31, 32, 34, 46]. Recently, the evolutionary fate of plastid-encoded *rps7*, *rpl20*, *rpoA*, *ycf1*, and *ycf2* have also been documented [26].

Although the Hypericaceae is one of the largest families of Malpighiales, complete plastome sequence has been reported for only one species, *Cratoxylum cochinchinense,* which belongs to the tribe Cratoxyleae (Hypericaceae); comparative genomic analysis for plastome evolution is very limited. In this study, we generated the complete plastome of *H. ascyron*, representing the first sequenced member of the tribe Hypericeae. The *H. ascyron* plastome organization is characterized, including functional replacements of five plastid genes to the nucleus. In addition, we analyzed *H. acyron* and nine published Malpighiales plastomes to examine patterns of plastome organization and highlighted nucleotide substitution rates within the clusioid of the Malpighiales.

## Results

### Plastome size and content of *Hypericum ascyron*

**Fig. 1:**
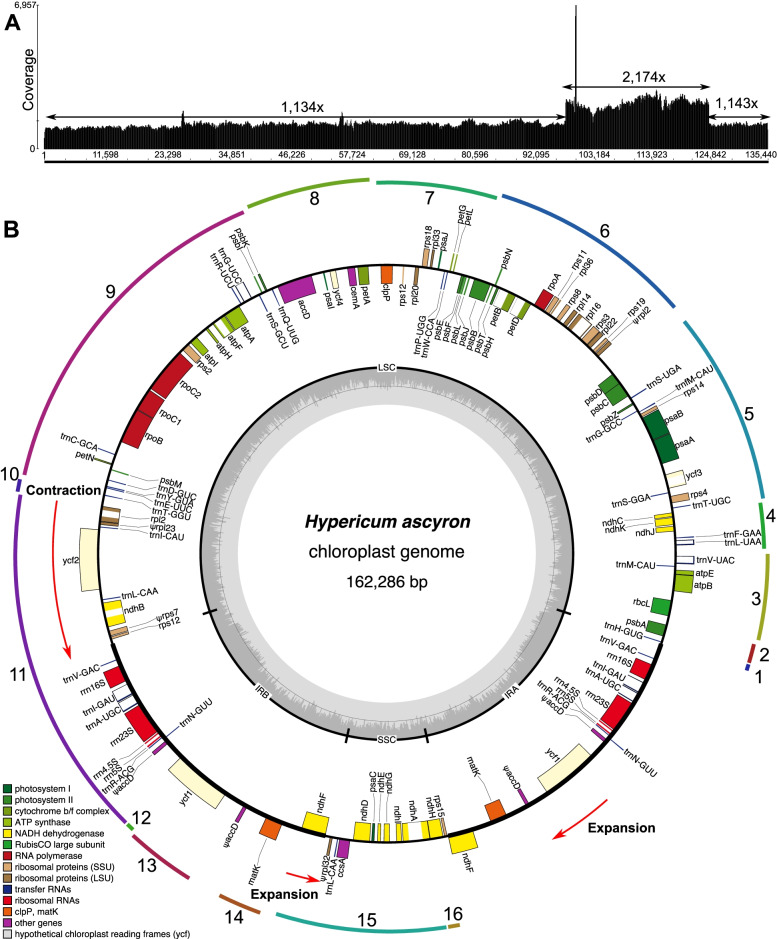
Map of the plastid genome of *Hypericum ascyron*. **A** Graph showing the base per base depth of the sequencing coverage across the *H. ascyron* plastome with one inverted repeat (IR) region. **B** Genes on the inside and outside of each map are transcribed in the clockwise and counterclockwise directions, respectively. The thick lines on the plastid map indicate the inverted repeats (IRA and IRB) that separate the genome into large and small single-copy regions. Ψ denotes a pseudogene. Red arrows indicate expansion events. Colored arcs on the outside of the map correspond to the locally collinear blocks inferred by Mauve (see Fig. 4)

Illumina sequencing produced 32,049,446 PE raw reads, providing deep coverage (> 1,100 ×) for the *H. ascyron* plastome (Fig. 1A). The assembled plastome of *H. ascyron* exhibits a circular molecule with two copies of IRs of 26,846 bp, separating the LSC and SSC regions of 97,542 and 11,052 bp, respectively (Fig. 1B, Table S1). IR regions of *H. ascyron* are general in size relative to the other clusioid clade plastomes analyzed, however, the LSC is the largest and the SSC is the smallest SSC among them (Table S1). The largest number of repeat pairs (46) was found in *H. ascyron* within 10 Malpighiales plastomes analyzed (Table S1). The fewest repeat pairs (6) were found in the *Sauvagesia rhodoleuca* and *Ctenolophon englerianus* (Table S1). *Hypericum ascyron* contains a higher number of repeats, except a repeat smaller than 50 bp (Figure S1). Repeats larger than 500 bp were only present in *H. ascyron* (Figure S1).

The *H. ascyron* plastome contained a set of genes encoding 74 proteins, 29 tRNAs and four rRNAs (Fig. 1, Table S1). The translation initiation factor A (*infA*), the ribosomal protein subunit S16 (*rps16*), and the tRNA^Lys^ (*trnK*-UUU) genes were absent in the plastome. In addition, the ribosomal protein subunit S7 (*rps7*), L23 (*rpl23*), and L32 (*rpl32*) genes appeared to be pseudogenes due to frameshift indels or internal stop codons. The sequences of three pseudogenes were confirmed by Sanger sequencing. We found that the *H. ascyron* plastome had lost both introns in *clpP*, the *cis*-spliced second intron in *rps12*, the *rpoC1* intron, and the second intron in *ycf3*.

**Fig. 2:**
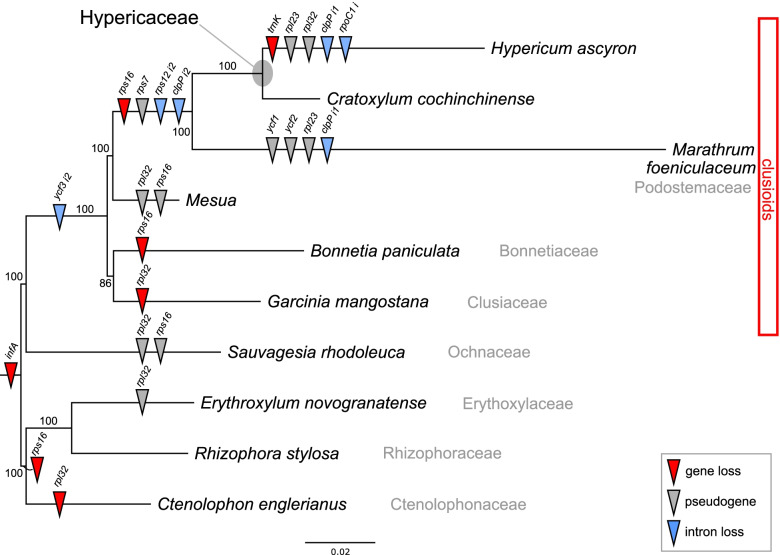
Phylogenetic distribution of gene/intron content (loss and gain) among the analyzed 10 Malpighiales

The phylogenetic distribution of gene and intron content across the selected 10 Malpighiales plastomes showed that the events of gene and intron losses were species- and lineage-specific (Fig. 2). While all analyzed plastomes lacked the *infA*, the phylogenetic distribution of the *rpl32* and *rps16* indicated that the loss or pseudogenization of the two genes occurred independently in the selected Malphigales plastomes. All species in the clusioid lineage were missing the second intron of the *ycf3* gene. *Hypericum ascyron*, *C. cochinchinense*, and *Marathrum foeniculaceum* shared the losses of *rps16*, the second *clpP* intron, and the *cis*-spliced second intron of *rps12* and the pseudogenization of *rps7*. Among the Malphigales species analyzed, the absence of the *trnK*-UUU gene and the *rpoC1* intron was unique to *H. ascyron*.

### Identification of gene transfer and substitution

**Fig. 3:**
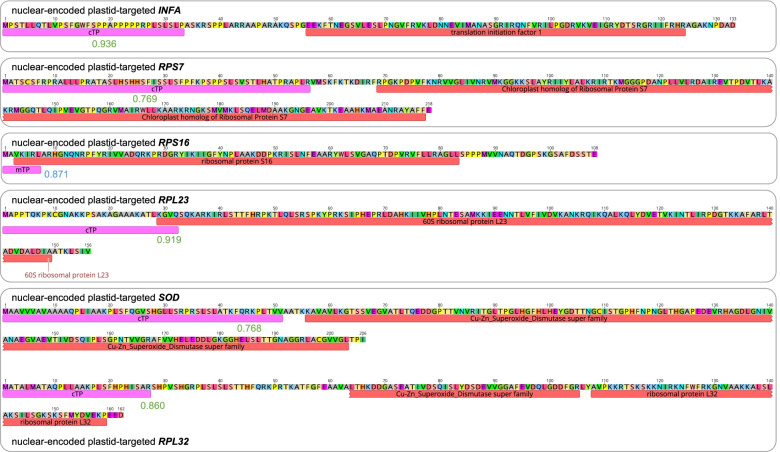
Amino acid sequence of nuclear-encoded plastid-targeted genes from *Hypericum ascyron*. Red boxes indicate the conserved domains, and pink boxes in the N-terminus indicate a transit peptide

Our results showed that the *H*. *ascyron* plastome lacked five protein-coding genes, *infA*, *rps7*, *rps16*, *rpl23*, and *rpl32*. To identify the functional replacement of these genes to the nucleus, we performed “tblastn” searches against the *H*. *ascyron* transcriptome data using the amino acid sequences of each gene from *Amborella trichopoda.* Nuclear transcripts of only two genes, *INFA* and *RPS7*, were detected in the *H*. *ascyron* transcriptome (Fig. 3). Further, we found that predicted ORFs of two transcripts had extended amino acids upstream from the conserved domain, and that TargetP predicted a plastid transit peptide with high probability (*INFA*: 0.936 and *RPS7*: 0.769) (Fig. 3). To test the potential gene substitution by a nuclear homolog, we queried the amino acid sequences of the *Medicago truncatula RPS16* and the spinach *RPL23* against the *H*. *ascyron* transcriptome. The tblastn searches using nuclear the homologs of *RPS16* and *RPL23*, two transcripts of each were identified (Figure S2A and B). Phylogenetic analysis of the nuclear-encoded *RPS16* copies from *H. ascyron* and five other Malphigales with two *Medicago* copies suggested two different origins, although both were predicted to contain a mitochondrial transit peptide (Figure S2A). In regard to *RPL23*, only one transcript included a plastid transit peptide (0.919) (Figure S2B).

To identify the nuclear-encoded *RPL32*, we queried the amino acid sequence of the *SODcp-RPL*32 chimeric gene from *Passiflora contracta* against the *H*. *ascyron* transcriptome. Two distinct transcripts were identified; one contains the conserved domain of “*Cu–Zn Superoxide Dismutase superfamily*” involving a plastid transit peptide (0.768) and the other contains the conserved domain of “*ribosomal protein L32*” and a plastid transit peptide (0.860) with a partial domain of “*Cu–Zn Superoxide Dismutase superfamily*” (Fig. 3). Amino acid sequence alignment of these two copies was divergent with low identity (23.9%) (Figure S2C). Compared with the available *SODcp-RPL*32 chimeric genes from Malpighiales species, the divergent pattern was similar to *Populus trichocarpa* (Figure S2C).

In addition, we also found the evidence of the functional replacement of these five genes to the nucleus in *H. perforatum* (Figure S3).

### Structural evolution of the *H. ascyron* plastome

**Fig. 4:**
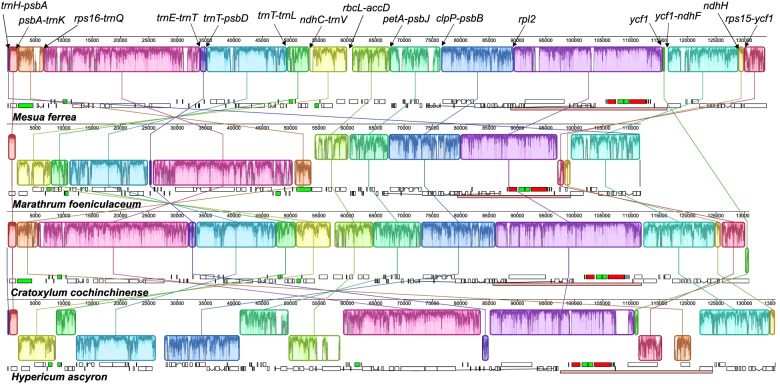
Structural alignments of plastomes from *Hypericum ascyron* with three related species using Mauve. The colored blocks represent collinear sequence blocks shared by all plastomes. Blocks drawn below the horizontal line indicate sequences found in an inverted orientation. Individual genes and strandedness are represented below each genome block. Only one copy of the inverted repeat (IR) is shown for each plastome and pink boxes below each plastome block indicate its IR

The *H. ascyron* plastome displayed dynamic changes, including multiple inversions, rearrangement, and IR boundary shifts, compared with the *Mesua ferrea* as an ancestral plastome structure (Fig. 4). Mauve alignment identified 16 locally collinear blocks (LCBs), suggesting seven inversions with 15 breakpoints: *trnH-psbA*, *psbA-trnK*, *rps16-trnQ*, *trnE-trnT*, *trnT-psbD*, *trnT-trnL*, *ndhC-trnV*, *rbcL-accD*, *petA-psbJ*, *clpP-psbB*, *rpl2*, *ycf1*, *ycf1-ndhF*, *ndhH*, and *rps15-ycf1* (Fig. 4). Among them, one inversion in the *H. ascyron* IR region was associated with IR expansion (Figs. 1 and 4). Furthermore, we observed that nine regions (LCB 3, 4, 5, 6, 7, 8, 9, 10, and 14) were relocated because of genome rearrangements, including inversions (Figs. 1 and 4). Among them, a notable gene relocation was *matK* gene, which transferred from the LSC region into the IR region (Fig. 1). The loss of the *trnK*-UUU gene seems to be correlated with this event, leaving only 655 bp of the 5’ *trnK* intron with 73.1% identity (Figure S4A).

The structure of the *H. ascyron* plastome also has changed with IR boundary shifts in relation to the *M. ferrea*, generating multiple relocated genes (Fig. 4). For example, contraction (13 kb) at the LSC/IR_A_ boundary excluded eight genes, from *rpl2* to *rps12*, and two expansions at the IR_B_/SSC and IR_A_/SSC have resulted in the duplication of two genes, full copies of *ndhF* and *ycf1* (Fig. 1).

We found that the partial sequence (371 bp) of the C-terminal region of *rpl2* was located upstream of the *rps19* gene (Figure S4B). The original copy of *rpl2,* downstream from *rpl23,* was truncated at the C-terminal (113 bp), but the conserved domain of ribosomal protein L2 was intact. The partial sequence was adjacent to the breakpoint and truncated original copy, suggesting the phenomenon was likely associated with the inferred inversion. In addition to the *rpl2*, the *H. ascyron* plastome contained four partial copies (513 bp) of surrounding the C-terminal portion of the *accD* (two are duplicated in the IR region) located in the IR region, with 96.7% identity (Figure S4C). The fragment next to the second *accD* copy is similar to a fragment located upstream of *psbD* gene, with 97.1% identity (Figure S4D).

In regard to an intact *accD*, its ORF in *H. ascyron accD* (3,204 bp) was highly expanded compared to the three related species (*C. cochinchinense*: 1,563 bp, *M. foeniculaceum*: 1,779 bp, and *M. ferrea*: 1,512 bp). The length of insertions in *H. ascyron accD* gene was confirmed by Sanger sequencing. Amino acid alignment of the four copies showed that *C. cochinchinense*, *M. foeniculaceum*, and *H. ascyron* contained amino acid insertions (Figure S5). Among them, we detected an interruption in the conserved domain of the *H. ascyron accD* gene, splitting the domain into two regions by a large insertion of 736 amino acids (Figure S5). The inserted regions in the *H. ascyron accD* gene was identified as multiple irregular amino acid repeats (Figure S6). However, the *accD* in *C. cochinchinense* and *M. foeniculaceum* contained only a small fraction of amino acids in the conserved domain (Figure S5)*.*

The *H. ascyron* plastome contained a *clpP*-like ORF (1,074 bp), which was also expanded compared to the coding region of the *M. ferrea clpP* gene (591 bp). The nucleotide sequences of the *clpP* coding regions were highly divergent with low identity match between *H. ascyron* and the other three species, ranging from 36.3% to 42.7% (Figure S6). Amino acid alignment of the four copies showed that the *H. ascyron clpP* gene contained small insertions of amino acids at the N- and C-terminal regions (Figure S7).

### Elevated substitution rates in the *H. ascyron* plastome

**Fig. 5:**
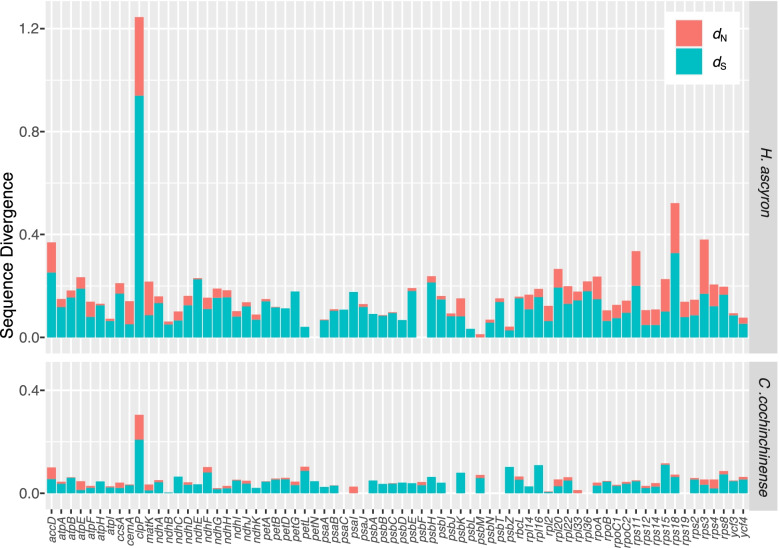
Variation in sequence divergence among *Hypericum ascyron* and *Cratoxylum cochinchinense* plastid protein-coding genes

The nonsynonymous (*d*_N_) and synonymous (*d*_S_) substitution rates of the *H. ascyron* plastid genes were 4.8 times and 3.2 times significantly higher than that of the *C. cochinchinense* plastid genes, respectively (Wilcoxon rank-sum test, *p* < 0.0001; Figure S8). The *H. ascyron* exhibited elevated substitution rates in most of the individual plastid genes in comparison with that in *C. cochinchinense*, showing that the average substitution values in *H. ascyron* were 6.4 times higher for *d*_N_ and 3.7 times higher for *d*_S_ than that in *C. cochinchinense* (Fig. 5). In particular, the *accD*, *clpP*, *rps3*, *rps11*, and *rps18* genes showed accelerated substitution rates compared with the *C. cochinchinense* (Fig. 5).

**Fig. 6:**
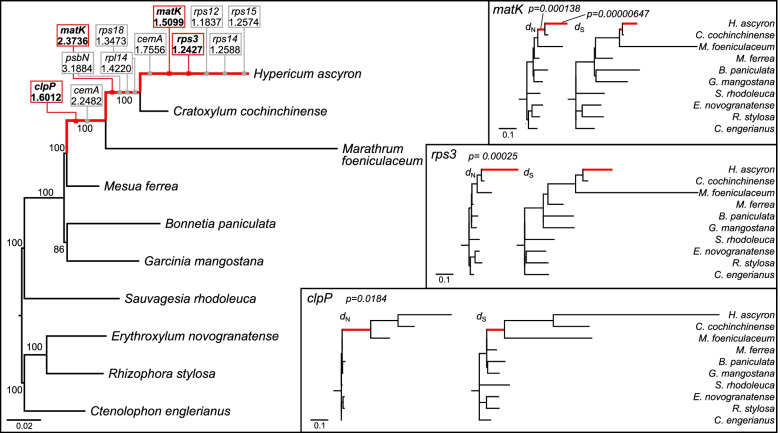
Plastid sequence divergence among selected Malpighiales. In the ML tree on the left, genes were selected to span the ratios of nonsynonymous to synonymous substitution rates greater than one on the branches related to *Hypericum ascyron*. Phylograms (right) of concatenated genes depicting nonsynonymous (*d*_N_) and synonymous (*d*_S_) sequence divergence for three individual genes are shown. Genes that show positive selection are selected. Red branches indicate genes with significantly higher *d*_N_/*d*_S_ values. All trees were drawn to the same scale

Lineage-specific accelerations of *d*_N_ and *d*_S_ substitution rates were detected within the three species that experienced structural changes in *accD* and *clpP* (Fig. 6, Figures S7 and S9). By observing the three branches related to *H. ascyron,* we identified that the *d*_N_/*d*_S_ values for the *cemA*, *clpP*, *matK*, *psbN*, *rpl14*, *rps3*, *rps12*, *rps14*, *rps15*, and *rps18* genes were greater than one (Fig. 6). Likelihood ratio tests (LRTs) indicated that the branches, leading to *H. ascyron/C. cochinchinense/M. foeniculaceum* with regard to *clpP,* leading to *H. ascyron/C. cochinchinense* with regard to *matK,* and *H. ascyron* terminal with regard to *matK* and *rps3*, were significantly different (*p* < 0.05 after Bonferroni correction, Fig. 6), indicating positive selection.

## Discussion

In this study, we first generated the complete plastome of *H. ascyron* from the tribe Hypericeae (Hypericaceae). The size of *H. ascyron* plastome at 162,286 bp is close to the size of *C. cochinchinense,* which belongs to the tribe Cratoxyleae (Hypericaceae). However, the complete *H. ascyron* plastome exhibited extreme changes in gene and intron content, organization, and in the rate of sequence divergence compared with the other members of the same family. Our results provide insights for improving the understanding of plastome evolution among the family.

### Evolutionary fate of the plastid gene losses in the *H. ascyron* plastome

The *H. ascyron* plastome lacked five protein-encoded genes (*infA*, *rpl23, rpl32, rps7,* and *rps16*), suggesting the activation of nuclear-encoded genes instead of the plastid genes. The phylogenetic distribution of the analyzed plastomes showed that the gene loss and pseudogenes are species- or lineage-specific (Fig. 2). These results suggest that functional replacement of plastid genes to the nucleus has occurred three times in the common ancestor of order Malpighiales (*infA*) or *H. ascyron/C. cochinchinense/M. foeniculaceum* (*rps16* and *rps7*), and in *H. ascyron* (*rpl23* and *rpl32*)*.* Many studies have shown that plastid-encoded gene loss or pseudogenes in plastids occur after successful functional replacement to the nucleus. For example, intracellular gene transfer to the nucleus has been documented in angiosperms, including *infA* in Ranunculaceae [24, 25], *rps7* in *Passiflora* [26], and *rpl32* in Ranunculaceae [24, 25, 33], Rhizophoraceae and Salicaceae [31, 32]. With regard to *rpl23* and *rps16*, gene substitution by the nuclear-encoded homologs have been reported: *rpl23* in spinach [29] and *Geranium* [30], *rps16* in *Lupinus* [35], *Medicago* and *Populus* [34], *Passiflora* [26], and Ranunculaceae [25, 33]. However, in Malpighiales, *rpl32* is acquired by alternative splicing of the nuclear-encoded Cu–Zn superoxide dismutase (SOD) gene, which targets plastid [32]. This suggests that the chimeric gene occurred in the common ancestor of the order Malpighiales [31]. Our results also provide clear evidence for potential functional replacements of the five plastid genes by gene transfer to the nucleus (*infA*, *rpl32,* and *rps7),* and substitution (*rpl23* and *rps16*) to the nucleus in *H. ascyron* (Fig. 3). All nuclear-encoded plastid-targeted *INFA*, *RPS7*, *RPS16*, *RPL23*, and *RPL32* copies of *H. ascyron* contain transit peptides at N-terminal acquired by a novel, exon shuffling of an existing nuclear-encoded gene for plastids, and substituted by nuclear-encoded homologs. With regard to *RPL32*, two highly divergent copies suggested that *H. ascyron* underwent subfunctionalization by duplication rather than alternative splicing. Similar patterns that occur sub-functionalization are observed in *Populus* and *Salix*, whereas mangroves (*Bruguiera gymnorrhiza*) contain a chimeric gene via an alternative splicing [31, 32]. Shrestha et al. (2020) showed that the transit peptide of *RPS7* was acquired by exon shuffling of a nuclear-encoded plastid-targeted thioredoxin m-type gene in *Passiflora*. However, the transit peptide acquisition of *H. ascyron RPS7* is unclear from the present data. For a comprehensive understanding of the evolutionary fate of the five plastid genes within this family and the clusioid clade, additional nuclear genomic and transcriptomic data from other species will be required.

### Structural variation in *H. ascyron* plastome

Multiple genomic rearrangements occurred in *H. ascyron* plastome, generating a number of gene relocations. Rearrangements promoted by homologous recombination between repeats has been proposed [47]. The number of repeat pairs in the *H. ascyron* plastome suggested that the repeats may facilitate rearrangements. Moreover, partial sequences of the *accD* throughout the LSC and IR regions can also rearrangements in *H. ascyron* plastome as repeated elements. In addition, the location of the *matK* as a free-standing gene, which was transferred into the IR region, suggested that genomic changes in *H. ascyron* involved multiple evolutionary mechanisms. Additional plastome sequences from other *Hypericum* species are needed to investigate the genome rearrangements including relocation of the *matK*.

IR boundary shifts (contraction and expansion) also affected the structure of the *H. ascyron* plastome, including gene reduction and duplication. For example, one copy of the *rpl2*, *rpl23*, *trnI*-CAU, *ycf2*, *trnL*-CAA, *ndhB*, *rps7*, and *rps12* genes are completely lost, whereas the *ndhF* gene is completely duplicated. Two cases of duplication, *matK* and partial of *accD*, in the *H. ascyron* plastome are likely due to a combination of rearrangements and IR expansion. The IR contraction and expansion generated the longest LSC and the smallest SSC among the analyzed Malpighiales, although IR size was median size (Table S1). The IR boundary shifts have occurred multiple times across angiosperm plastomes, which is caused by double-strand break model [48, 49].

### Substitution rate variation in *H. ascyron* plastome

A gene-specific rate increase compared with that in the *C. cochinchinense* plastid genes was observed in *H. ascyron* plastid genes (Fig. 5). Previous studies have shown a positive correlation between substitution rates and genomic rearrangements [7, 8, 14]. Multiple genomic changes including inversions in the *H. ascyron* can influence the elevated substitution rates in the plastid genes (Fig. 4). In addition, a positive effect of structural evolution on substitution rates of the *H. ascyron accD* and *clpP* genes also were identified. Highly divergent, intronless *clpP* gene in *H. ascyron* plastome was detected with greatly higher *d*_N_ and *d*_S_ values compared with the analyzed species. Likewise, correlation between elevated substitution rates and the loss of introns of the *clpP* gene has been observed in some angiosperm lineages, *Geranium* [22], legume [50], and *Silene* [51]. Possible mechanisms of intron loss have been proposed: direct genomic deletion, exonization of intron, and retroprocessing [52]. The phylogenetic distribution showed that the loss of the second intron occurred in the common ancestor of *H. ascyron, C. cochinchinense* and *M. foeniculaceum* followed by loss of the first intron independently in *H. ascyron* (Fig. 2), suggesting that different mechanisms may have been activated for each intron loss. In particular, our analysis showed evidence of positive selection on the branch leading to *H. ascyron/C. cochinchinense/M. foeniculaceum* (Fig. 6). In the plastid *accD*, the insertion of amino acids is also correlated with the accelerated substitution rates (Figure S9). Interruption of the *accD* was identified in *Geranium* [22] and *Lamprocapnos* [10] and the genes exhibit elevated substitution rates, although the patterns of insertion are different. However, the force driving the positive selection on the *H. ascyron* terminal branch with regard to *rps3* is unclear.

In the plant, maturase K (*matK*) is located within the intron of *trnK*-UUU in the plastome. This *matK* product is essential for the splicing of group II introns, including *cis*-spliced introns of *atpF*, *rpl2*, *rps12*, *trnV*-UVC, *trnI*-GAU, *trnA*-UGC, and *trnK*-UUU. The loss of the *trnK*-UUU gene has been found in *Cuscuta* [53], *Epifagus* [54], *Taxillus* [55] and *Erodium* [14]. In *H. ascyron*, the surrounding regions of the *trnK*-UUU exons were lost and the *matK* was retained as a free-standing gene in the plastome. The *H. ascyron* plastome contained the five introns, although the *cis*-spliced *rps12* and *trnK*-UUU introns were lost, indicating the functional necessity of the *matK*. The *d*_N_/*d*_S_ values of the branches, leading to *H. ascyron*/*C. cochinchinense* and *H. ascyron* terminal were greater than one, and the LRT suggested that the *matK* gene underwent positive selection. The forces driving the positive selection in the *H. ascyron matK* are likely associated with the loss of *trnK*-UUU and relocation. However, *C. cochinchinense matK* is encoded within the intron of *trnK*-UUU in its plastome and does not relocate. The plastid *matK* in the lineage leading to *H. ascyron*/*C. cochinchinense* is likely to experience altered selection pressures. A complete understanding of these patterns of higher substitution rates requires sufficient samples from this family.

## Conclusions

The plastome sequence of *H. ascyron*, a member of the tribe Hypericeae, provides new insights into the plastome evolution within Hypericiaceae. The plastome exhibits a number of unusual phenomena, including genome rearrangements, gene and intron losses, and elevated substitution rates compared with the *C. cochinchinense* plastome. Nuclear transcriptome data provide clear evidence for functional replacements by gene transfers (*infA*, *rps7*, and *rpl32*) or substitution (*rps16* and *rpl23*) from the plastids to the nucleus.

## Methods

### DNA isolation, sequencing, assembly, and annotation

*Hypericum ascyron* was collected from Mt. Cheayak, Yeongcheon-si, Gyeongsangbuk-do, South Korea. The plant material used in this study was obtained from the wild and a voucher specimen was deposited in the Yeungnam University Herbarium (YNUH0202519 identified by SeonJoo Park). Experimental study on the plant, including collection of the material, comply with institutional, national, and international guidelines. Total DNA was isolated from fresh leaves (100 mg) using the DNeasy Plant Mini Kit (Qiagen Inc., GmbH, Germany). Total DNA was processed for genomic library preparation using the Hiseq2500 platform (Illumina, San Diego, CA, USA), generating 5.6 Gb of 2 × 150 bp paired-end (PE) reads from a 550 bp library. PE reads were used for de novo assembly using Velvet v1.2.10 assembler [56] with multiple *k*-mers ranging from 99 to 145 and expected coverage values (100, 200, 300, 400, and 500). To evaluate the depth of coverage, PE reads were mapped to the plastome with one IR region using Bowtie2 v2.2.6 [57]. The plastome was annotated using Geneious R11 v11.0.5 (https://www.geneious.com) with the protein coding genes of *Nicotiana tabacum* as a reference, and the open reading frame (ORF) of the plastome was evaluated. All tRNA genes were identified using tRNAscan-SE v2.0.3 [58] and ARAGON v1.2.38 [59]. The OGDraw v1.3.1 [60] was used to draw the circular plastome of *H. ascyron*. The annotated plastome sequence was deposited in GenBank (accession number MZ424306).

To check the assembly errors in the *accD*, *rpl23*, *rpl32*, *rps7* copies, polymerase chain reaction (PCR) was carried out with specific primer pairs (Table S2), using methods described previously [10]. The PCR products were purified using the Solg™ Gel & PCR extraction system (Solgent Co., Daejeon, South Korea) following the manufacturer’s protocol and sequenced using an ABI 3730xl DNA Analyzer (Applied Biosystems, California, USA) at Solgent Co. Repeats in the *accD* gene were identified using “Find Repeats” option in Geneious R11 with minimum repeat length of 20 bp.

### Validation of functional replacements of plastid genes to the nucleus

Total RNA was extracted from fresh leaves of *H. ascyron* using the HiGene™ Total RNA Prep Kit (ver. 2.0) (Biofact, Daejeon, South Korea) following the manufacture’s protocol. The RNA was sequenced using the Illumina HiSeq4000 platform with 2 × 150 bp PE reads. Error correction was performed using Rcorrector [61] with default parameters. To identify nuclear-encoded plastid-targeted genes, the transcriptome from *H. ascyron* was assembled using Trinity v2.11.0 [62] with the trimmomatic flag. Potential transcripts were identified using “tblastn” searches of the plastid-encoded genes (*infA*, *rps7, rps16, rpl23*, and *rpl32* from *Amborella trichopoda* plastome; NC_005086) and nuclear-encoded genes (*RPS16* from *Medicago truncatula*; AB365526, *RPL23* from *Spinacia oleracea*; Q9LWB5, *SODcp-RPL*32 from *Passiflora contracta*; MT259558) against the *H. ascyron* transcriptome. The open reading frames (ORFs) in the transcripts were determined and translated using Geneious R11 and subsequently were used as queries. TargetP v.1.1 [63] was used to predict transit peptides, and the NCBI Conserved Domain Database (CDD) was used to annotate the functional domain of proteins [64]. The published PE reads of *Hypericum perforatum* (SRR8438984) was also used. Transcriptome assembly and identification of the nuclear-encoded plastid-targeted genes were performed as described above.

### Comparative analyses

Plastome rearrangements in *H. ascyron* were compared with nine genera from nine families (Bonnetiaceae, Calophylleae, Clusiaceae, Ctenolophonaceae, Erythroxylaceae, Hypericaceae, Ochnaceae, Podostemoideae, and Rhizophoraceae) of Malphigales (Table S1) using Mauve v2.3.1 [65] in Geneious R11 with default parameters. Repetitive sequences were identified by performing “blastn” searches [66] of each plastome against itself with an e-value cutoff of 1e-10 after removing one copy of IR.

### Phylogenetic and substitution rate estimation

To reconstruct phylogenetic relationships among other Malphigales, 70 plastid protein-coding genes from the selected 10 plastomes were extracted (Table S3). The individual genes were aligned using the Translation Align-Maximum likelihood (ML)-based MAFFT v7.450 [67] in Geneious R11 and concatenated to a single alignment data set. The maximum likelihood (ML) tree was generated using IQ-TREE v1.6.2 [68] with an ultrafast bootstrap algorithm (1000 replicates). Nonsynonymous (*d*_N_) and synonymous (*d*_S_) nucleotide substitutions for all selected gene trees were calculated in PAML v4.8 [69] using the CODEML program with the F3 × 4 codon frequency mode. To test for branch sites under positive selection, we used the “adaptive BSREL” model [70] implemented in HyPhy v2.5 [71] using the Datamonkey server [72], which performs a series of likelihood ratio tests (LRTs) using the Holm-Bonferroni correction.

## Supplementary Information

**Additional file 1.**

## Data Availability

The data sets supporting the results of this article are included in additional files. Complete plastid genome and gene sequences are available in the GenBank (https://www.ncbi.nlm.nih.gov/nuccore/MZ424306, OM967034-OM967041).

## Abbreviations

*d*_N_: Number of substitutions per nonsynonymous site
*d*_S_: Number of substitutions per synonymous site
LRT: Likelihood ratio test
ORF: Open reading frame
LSC: Large single copy
SSC: Small single copy
IR: Inverted repeat
