# Supplementary material for: Gene loss, genome rearrangement, and accelerated substitution rates in plastid genome of Hypericum ascyron (Hypericaceae)
Source: BMC Plant Biol. 2022 Mar 23;22:135. doi: 10.1186/s12870-022-03515-x (PMC8941745; doi:10.1186/s12870-022-03515-x)
Supplement: Supplementary file 1 — Additional file 1. [file 12870_2022_3515_MOESM1_ESM.pdf]

Article title: Gene loss, genome rearrangement, and accelerated substitution rates in plastid genome of *Hypericum ascyron* (Hypericaceae)

Authors: Sivagami-Jean Claude, Seongjun Park, SeonJoo Park

Additional material

Figure S1. Repeat size and frequency in *Hypericum ascyron* plastome relative to nine other representative species.

Figure S2. Amino acid sequence alignment of the nuclear-encoded *RPS16, RPL23,* and *RPL32* copies of the *Hypericum ascyron* and outgroups.

Figure S3. Amino acid sequence of nuclear-encoded plastid-targeted genes from *Hypericum perforatum.*

Figure S4. Structural alignments of *Hypericum ascyron* plastome.

Figure S5. Amino acid sequence alignment of plastid-encoded *accD* including the insertion regions from four species.

Figure S6. Characterization of the plastid-encoded acetyl-CoA carboxylase beta subunit (*accD*) genes of *Hypericum ascyron*.

Figure S7. Amino acid sequence alignment of plastid-encoded *clpP* including the insertion regions from four species.

Figure S8. Box plots of the values of nonsynonymous and synonymous substitution rates for *Hypericum ascyron* (blue) and *Cratoxylum* (red) plastid genes.

Figure S9. Phylograms of plastid *accD* gene showing nonsynonymous (d_N) and synonymous (d_S) substitution rates.

Table S1. General characteristics of 10 Malpighiales plastomes.

Table S2. Primers used to confirm plastid *accD*, *rpl23*, *rpl32*, and *rps7* gene regions.

Table S3. Plastid genes include in the phylogenetic analysis.

Figure S1. Repeat size and frequency in *Hypericum ascyron* plastome relative to nine other representative species. Repeats were divided according to size: 50-99, 100-199, 200-299, 500-999 and greater than 1000 bp.

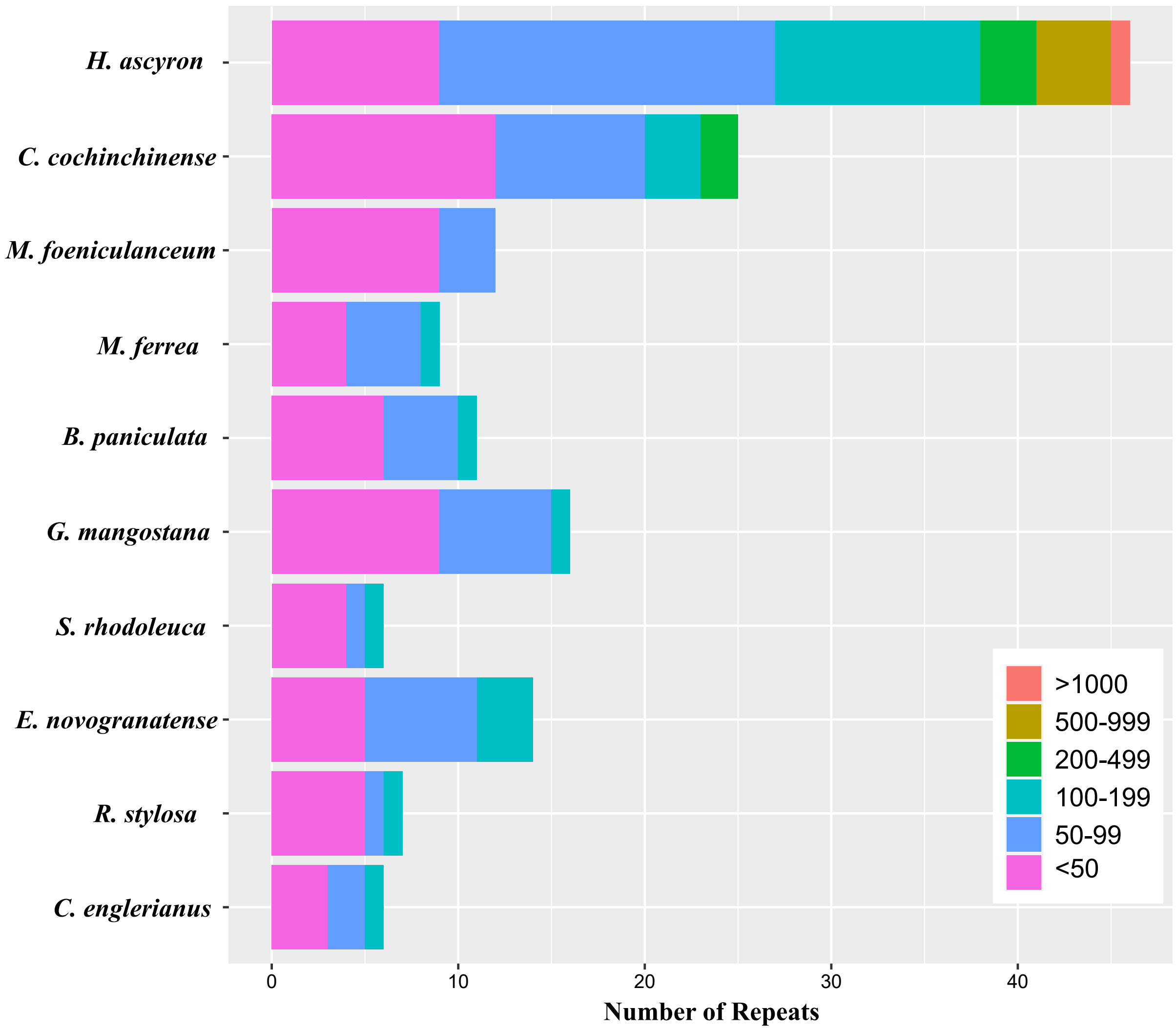

Figure S2. Amino acid sequence alignment of the nuclear-encoded *RPS16, RPL23,* and *RPL32* copies of the *Hypericum* and outgroups. A. Amino acid alignment of nuclear-encoded *RPS16* copies from *Hypericum* (above). Pink and red annotations indicate a transit peptide and a conserved domain of *RPS16* in *Hypericum*. Amino acid alignment of nuclear-encoded *RPS16* copies from *Hypericum* and related species with phylogenetic tree (below). The sequence logo shows the most conserved amino acid. Red box indicate a conserved domain. NUPT (green box) and NUMT (blue box) were determined by phylogenetic position of *Medicago rps16* genes. **B**. C. Amino acid alignment of nuclear-encoded *SODcp-RPL32* chimeric genes from *Hypericum* with related species. Pink and red boxes indicate a transit peptide and a conserved domain of each protein. The sequence logo shows the most conserved amino acid.

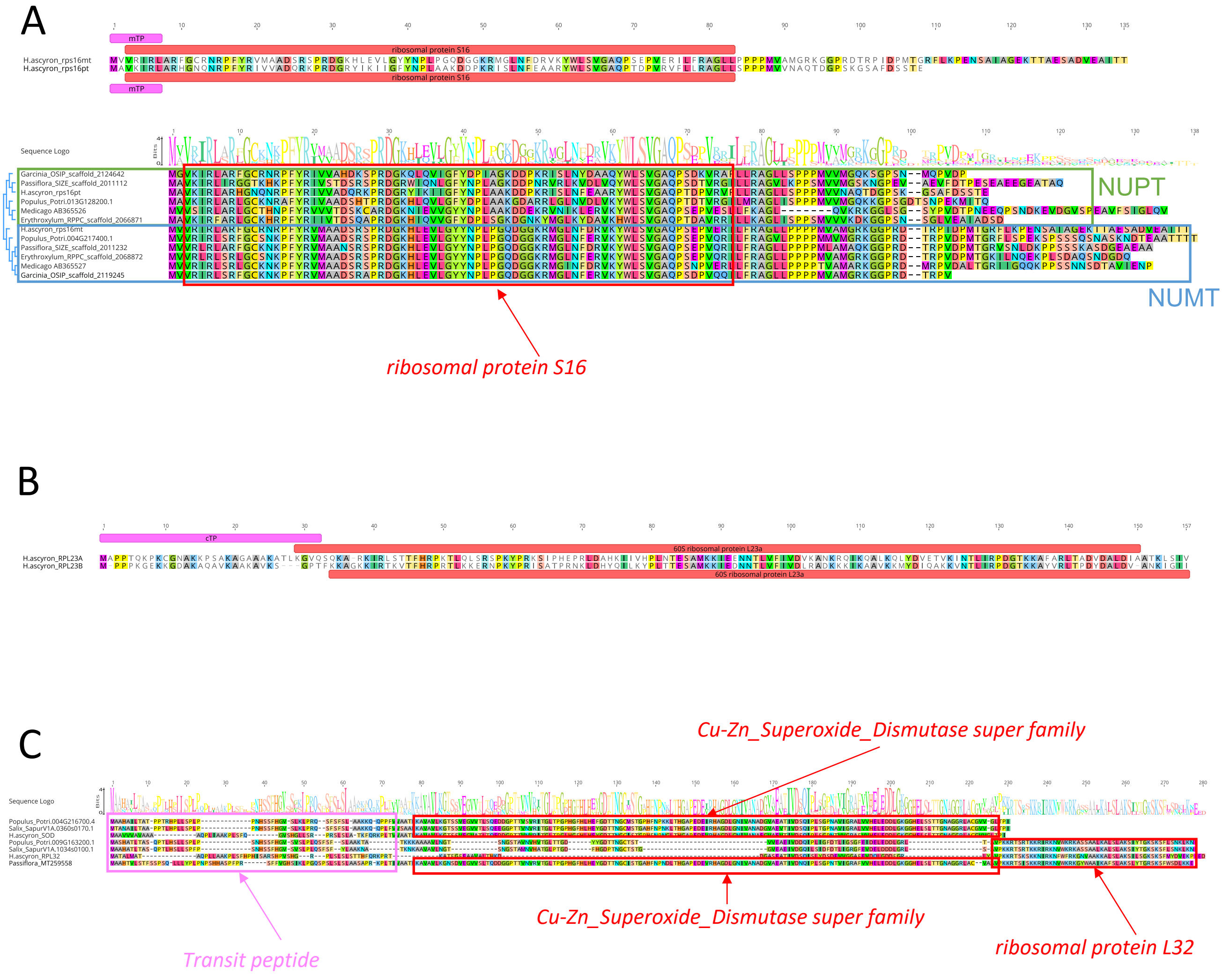

Figure S3. Amino acid sequence of nuclear-encoded plastid-targeted genes from *Hypericum perforatum*. Red boxes indicate the conserved domains, and pink boxes in the N-terminus indicate a transit peptide.

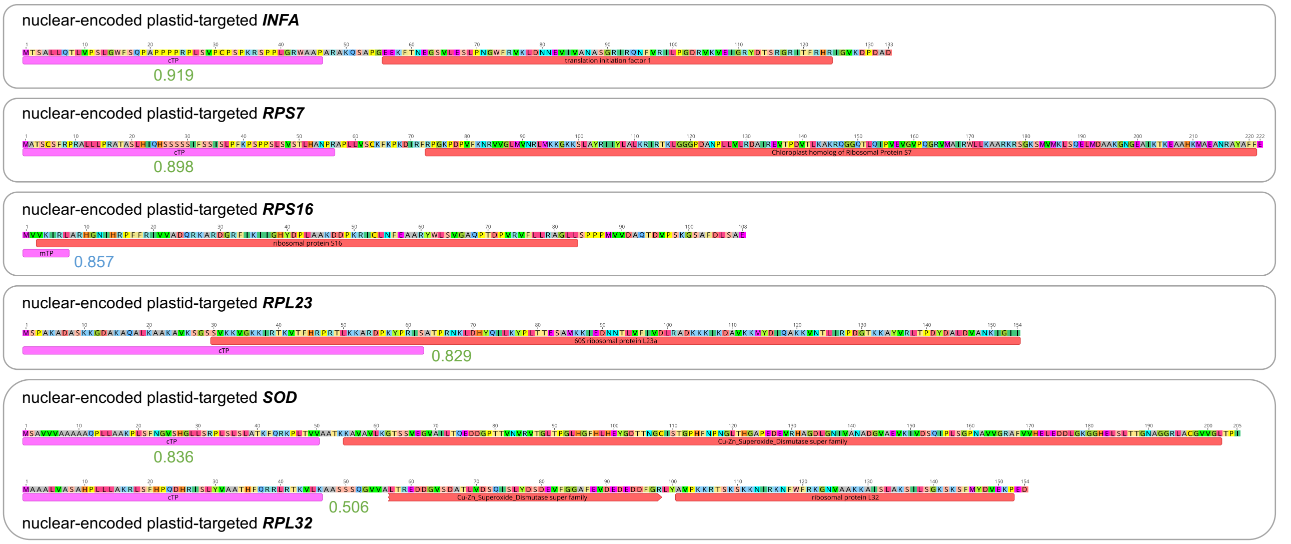

Figure S4. Structural alignments of *Hypericum ascyron* plastome.
The genes above and below the horizontal line correspond to the genes in Figure 1. Gray boxes indicate IR regions. Nucleotide sequence alignments of plastid *accD*, *rpl2*, *matK*, and interested regions. Green, orange, and yellow annotations indicate gene, blast hit, and coding regions.

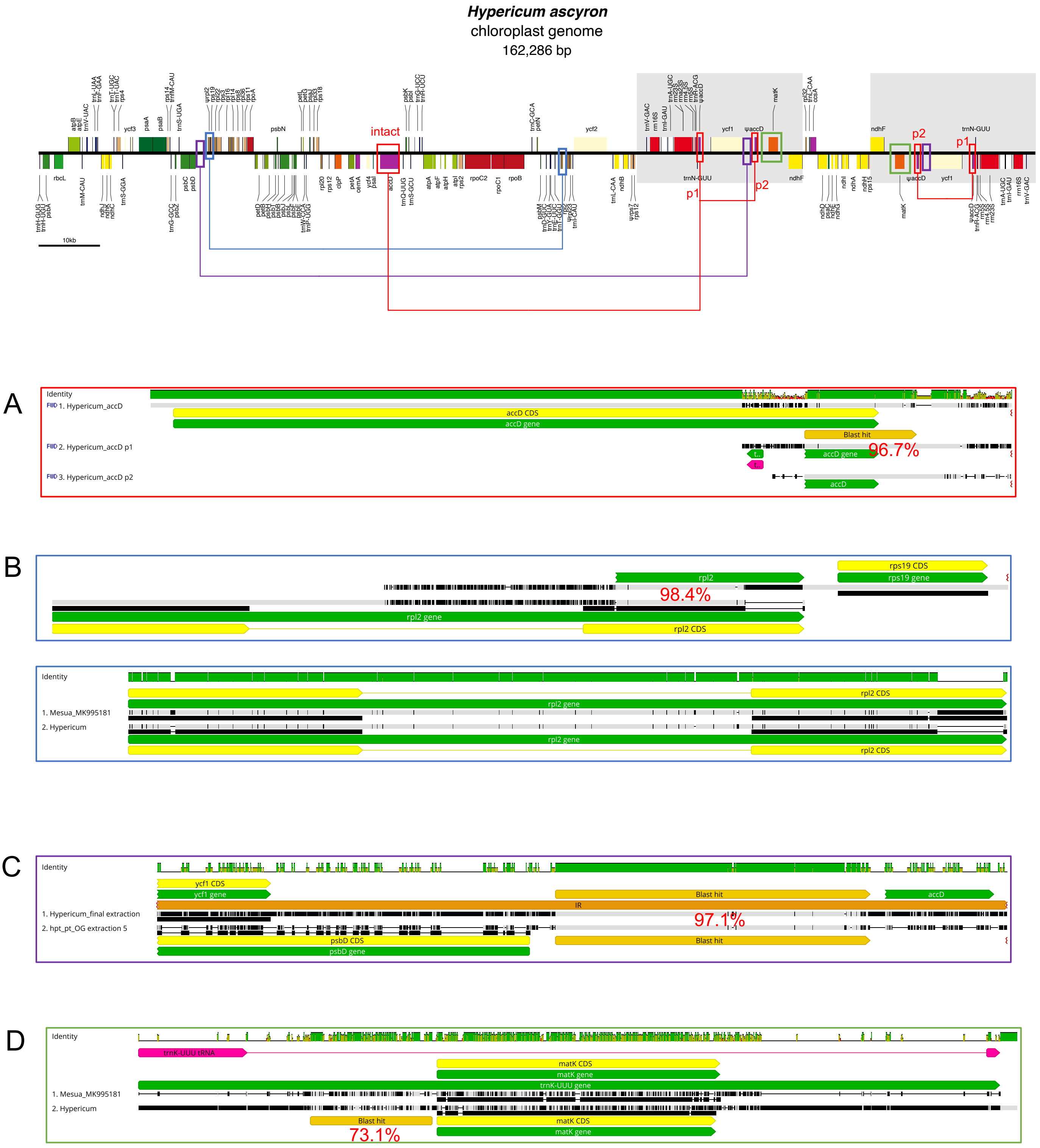

Figure S5. Amino acid sequence alignment of plastid-encoded *accD* including the insertion regions from four species. Red boxes indicate the conserved domain of acetyl-CoA carboxylase beta subunit. The sequence logo shows the most conserved amino acid.

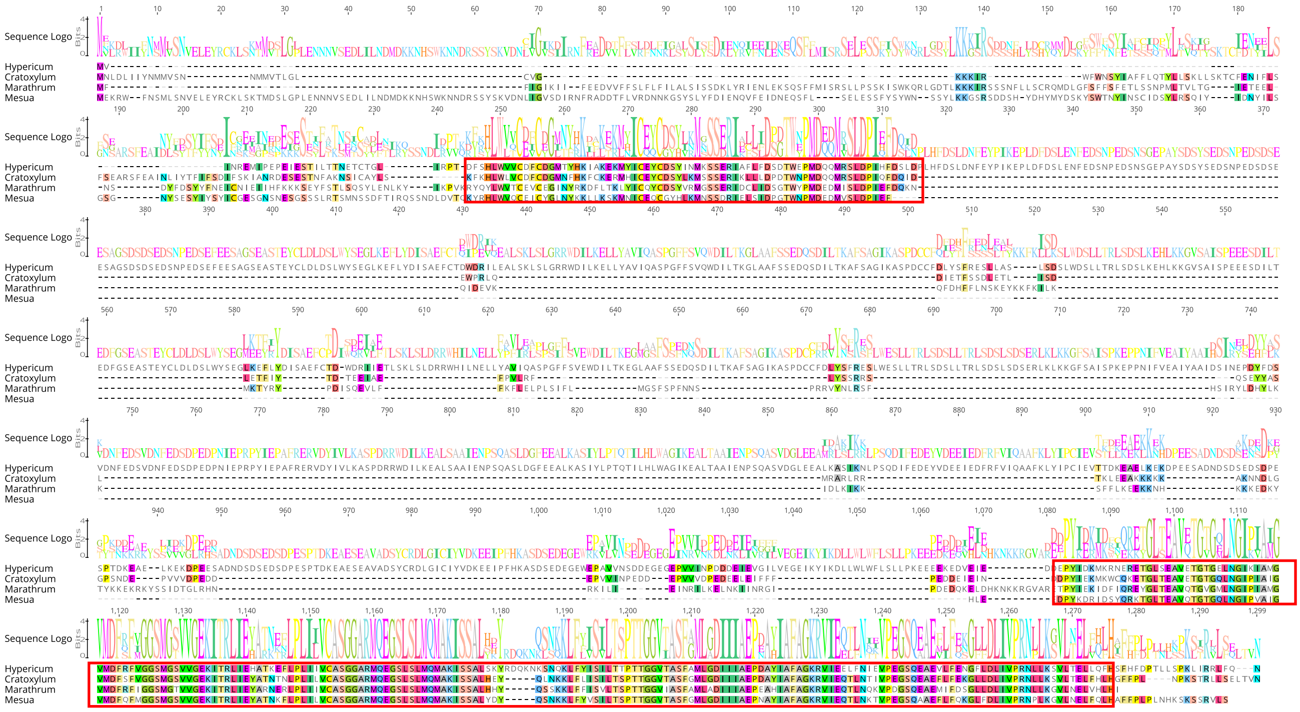

Figure S4. Characterization of the plastid-encoded acetyl-CoA carboxylase beta subunit (*accD*) genes of *Hypericum ascyron*. Schematic diagram of the *accD* gene. Pink box indicate a conserved domain of acetyl-CoA carboxylase beta subunit, which identified by the CDD at the NCBI. Identical repetitive sequences were marked with the same colored boxes.

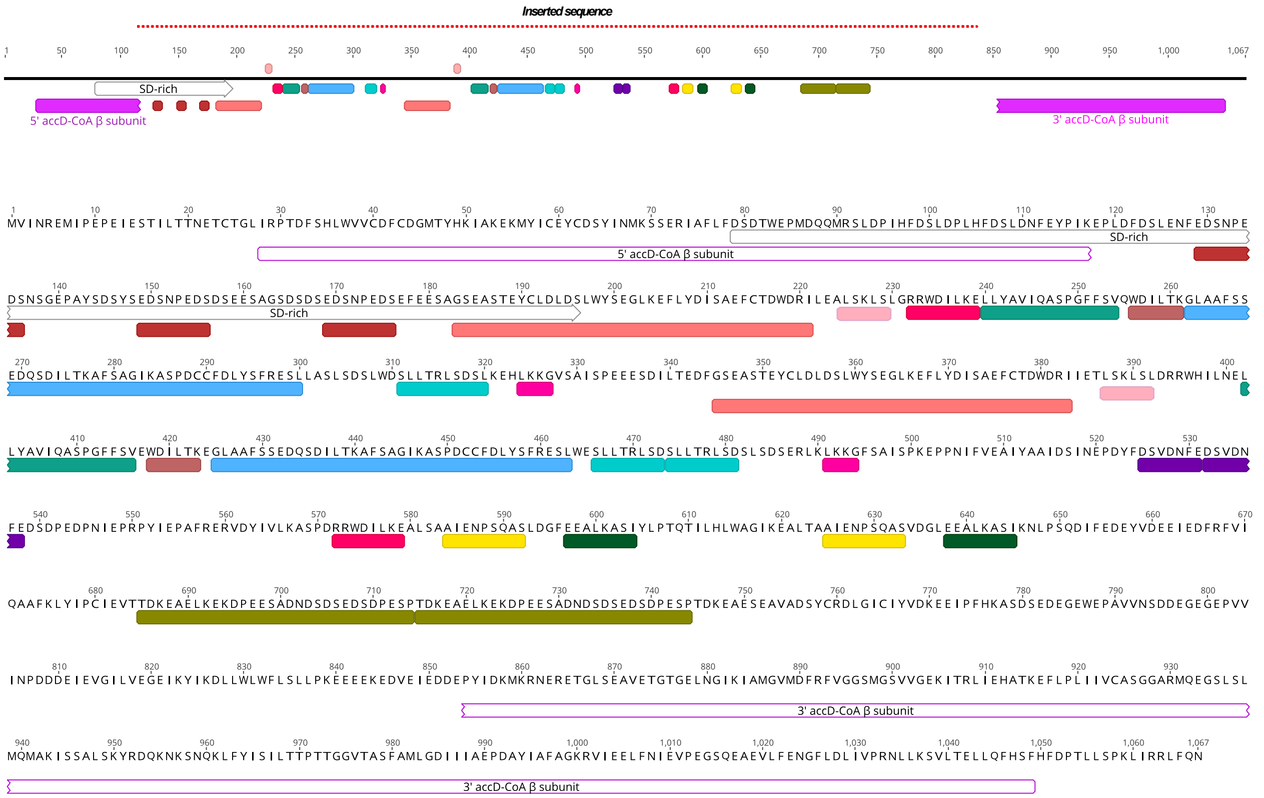

Figure S5. Amino acid sequence alignment of plastid-encoded *clpP* including the insertion regions from four species. Arrowheads indicate the positions of the first and second introns. The sequence logo shows the most conserved amino acid.

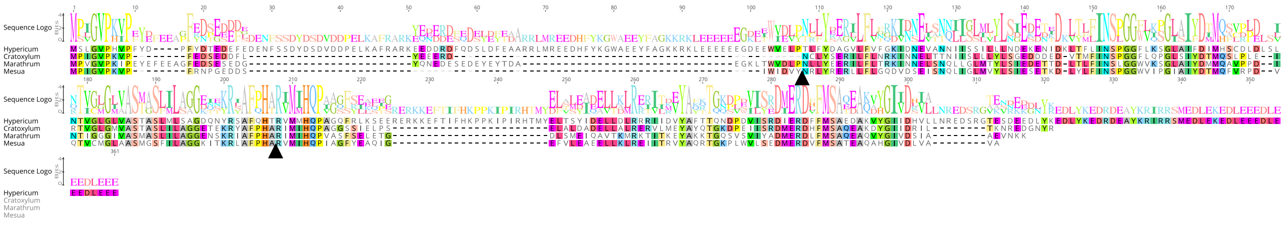

Figure S6. Box plots of the values of nonsynonymous and synonymous substitution rates for *Hypericum ascyron* (blue) and *Cratoxylum cochinchinense* (red) plastid genes. The box represents values between quartiles, the solid lines extend to the minimum and maximum values, outliers are shown as circles and horizontal lines in the boxes show the median values. Significance of fit was evaluated by Wilcoxon rank sum tests in the R package.

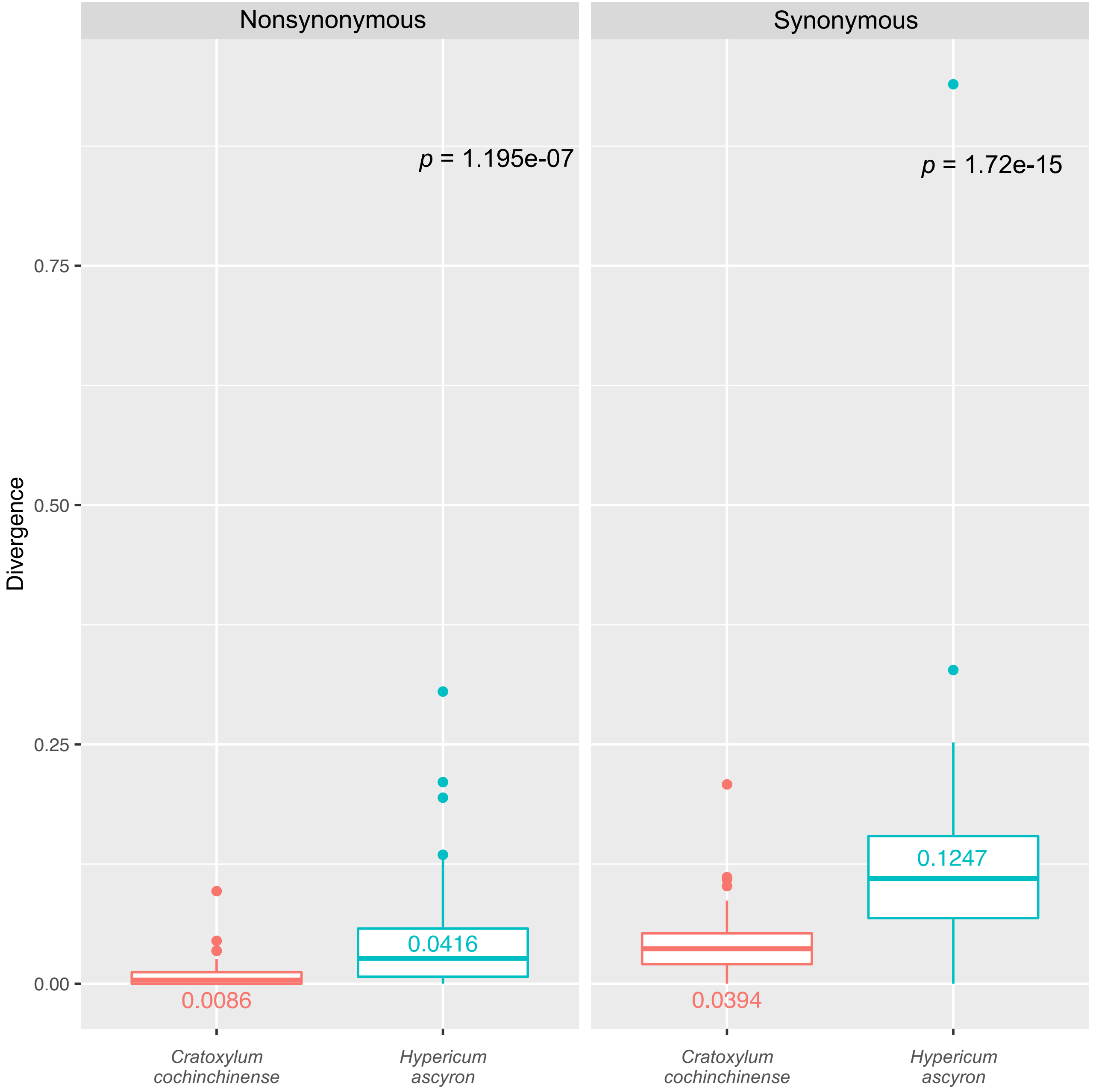

Figure S7. Phylograms of plastid *accD* gene showing nonsynonymous (d_N) and synonymous (d_S) substitution rates. Lightning symbols indicate the lineages with a disrupted *accD* gene

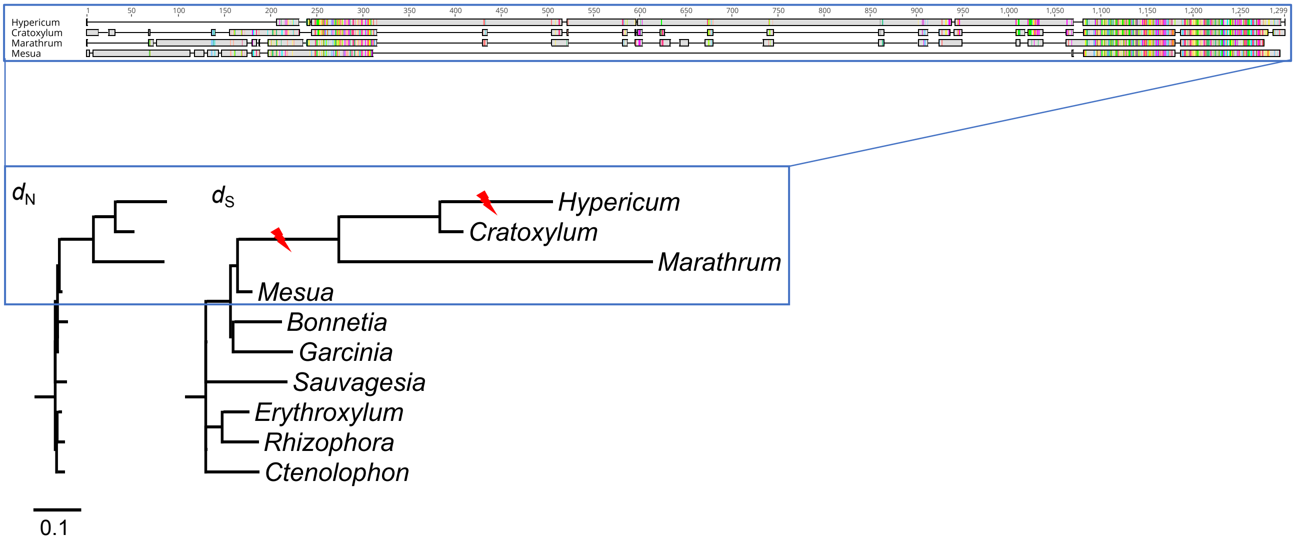

Table S1. General characteristics of 10 Malpighiales plastomes.

Family	Genus	Species	Genome size (bp)	LSC (bp)	IR(bp)	SSC(bp)	GC content (%)	Repeat pairs	Repeat portion	Accession number
Hypericaceae	*Hypericum*	*Hypericum ascyron*	162,286	97,542	26,846	11,052	37.40%	46	0.08	MZ424306
	Cratoxylum	*Cratoxylum cochinchinense*	157,103	85,664	26,272	18,895	36.20%	25	0.0183	MN399961
Podostemoideae	*Marathrum*	*Marathrum foeniculaceum*	131,600	79,502	19,916	12,266	35.10%	12	0.00667	MK995178
Calophylleae	*Mesua*	*Mesua ferrea*	161,494	88,784	27,614	17,482	36.40%	9	0.0061	MK995181
Bonnetiaceae	*Bonnetia*	*Bonnetia paniculata*	156,782	84,024	27,309	18,140	36.20%	11	0.00573	MK995182
Clusiaceae	*Garcinia*	*Garcinia mangostana*	158,179	86,458	27,009	17,703	36.10%	17	0.00943	NC_036341
Ochnaceae	*Sauvagesia*	*Sauvagesia rhodoleuca*	157,300	86,021	26,571	18,137	36.40%	6	0.00489	MW772237
Erythroxylaceae	*Erythroxylum*	*Erythroxylum novogranatense*	163,937	91,383	27,208	18,136	35.90%	14	0.007423	NC_030601
Rhizophoraceae	*Rhizophora*	*Rhizophora stylosa*	164,475	92,582	26,325	19,243	34.90%	7	0.00417	NC_042819
Ctenolophonaceae	*Ctenolophon*	*Ctenolophon englerianus*	161,553	89,386	27,469	17,237	36.00%	6	0.004079	NC_049158

Table S2. Primers used to confirm plastid *accD*, *rpl23*, *rpl32*, and *rps7* gene regions. Asterisk indicates a primer that used only Sanger sequencing.

PCR set	Forward	Sequence (5'->3')	Reverse	Sequence (5'->3')
accD	56192F	AACAGCCTCAGACAATCCCG	58702R	CTATGGGTGGTGTGCGACTT
	56975F*	AATGGTTTGGGTGGGAAGGT		
rpl23	86315F	ACGGCCTTTACCACAACGAT	87418R	ACTACGCCCTTGATCGTGAA
rps7	95752F	TCTTCTCTCCACCGGAACAA	96949R	GCTGTCGGAGTAAAGGGTCG
rpl32	124273F	TCGGAAAAAGTAGAAGCCCC	125461R	GCGGCTTGCTCCAAATCATA

Table S3. Plastid genes include in the phylogenetic analysis.

Category	Gene Type	Genes
Self-replication	Small ribosomal units	*rps2, rps4, rps8, rps11, rps12, rps14, rps15, rps18, rps19*
	Large ribosomal units	*rpl2, rpl14, rpl16, rpl20, rpl22, rpl33, rpl36*
	RNA ploymerase subunits	*rpoA, rpoB, rpoC1, rpoC2*
Photosynthesis genes	ATP Synthase	*atpA, atpB, atpE, atpF, atpH, atpI*
	NADH dehydrogenase	*ndhA, ndhB, ndhC, ndhD, ndhE, ndhF, ndhG, ndhH, ndhI, ndhJ, ndhK*
	Photosystem I	*psaA, psaB, psaC, psaI, psaJ, ycf3, ycf4*
	Cytochrome b6/f Complex	*petA, petB, petD, petG, petL, petN*
	Photosystem II	*psbA, psbB, psbC, psbD, psbE, psbF, psbH, psbI, psbJ, psbK, psbL, psbM, psbT, psbZ*
	Large subunit of rubisco	*rbcL*
other genes	Maturase	*matK*
	Protease	*clpP*
	Accetyl-CoA-corbpxylase subunit	*accD*
	Envelope membrane protein	*cemA*
	c-type cytochorme synthesis	*ccsA*
